# Magnetic assembly of transparent and conducting graphene-based functional composites

**DOI:** 10.1038/ncomms12078

**Published:** 2016-06-29

**Authors:** Hortense Le Ferrand, Sreenath Bolisetty, Ahmet F. Demirörs, Rafael Libanori, André R. Studart, Raffaele Mezzenga

**Affiliations:** 1 Complex Materials, Department of Materials, ETH Zurich, Zurich 8093, Switzerland; 2 Food and Soft Materials, Department of Health Sciences and Technologies, ETH Zurich, Zurich 8092, Switzerland

## Abstract

Innovative methods producing transparent and flexible electrodes are highly sought in modern optoelectronic applications to replace metal oxides, but available solutions suffer from drawbacks such as brittleness, unaffordability and inadequate processability. Here we propose a general, simple strategy to produce hierarchical composites of functionalized graphene in polymeric matrices, exhibiting transparency and electron conductivity. These are obtained through protein-assisted functionalization of graphene with magnetic nanoparticles, followed by magnetic-directed assembly of the graphene within polymeric matrices undergoing sol–gel transitions. By applying rotating magnetic fields or magnetic moulds, both graphene orientation and distribution can be controlled within the composite. Importantly, by using magnetic virtual moulds of predefined meshes, graphene assembly is directed into double-percolating networks, reducing the percolation threshold and enabling combined optical transparency and electrical conductivity not accessible in single-network materials. The resulting composites open new possibilities on the quest of transparent electrodes for photovoltaics, organic light-emitting diodes and stretchable optoelectronic devices.

## Abstract (web summary)

Transparent and electrically conducting flexible films are in high demand but production can be both time-consuming and expensive. Here, the authors report a method for assembling modified graphene flakes in controlled distributions within polymeric matrices by use of magnetic fields.

## Graphical abstract

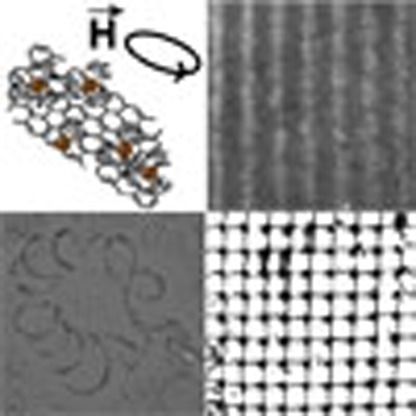

The increasing demand for optoelectronic tools from tissue-compatible biomedical devices for health monitoring to indium–tin-oxide-free electrodes for flexible solar cells has recently stimulated the search for cost-effective materials with comparable performance, but improved flexibility to replace the commonly used stiff and brittle components^1^,^2^,^3^,^4^. There is an extensive library of existing potential materials combining electrical conductivity and transparency, mostly based on substrate films coated with a mesh of nanocarbons or metal nanowires^5^,^6^. These materials present high electrical conductivity, corresponding to a sheet resistance down to 128 Ohm sq^−1^ with 95% transparency, while flexibility is provided to a certain extent by the polymeric substrate. However, bulk materials containing mixtures of the conductive elements and the flexible matrix present the advantage to provide more mechanical integrity over sandwich or layered structures, as well as the possibility to create more complex three-dimensional (3D) circuits. In addition, direct ink writing or 3D printing techniques can be applied to build on-demand materials with inks containing both the transparent support and the conductive elements^7^,^8^,^9^. Conductive and transparent polymers and blends based on poly(3,4-ethylenedioxythiophene) doped with polystyrene sulfonic acid (PEDOT:PSS) can reach transparency up to 87% combined with a conductivity of 1.35 S cm^−1^ for films of 1 μm thickness^10^. Yet, even in this case, good performances are typically recorded for ultrathin films and based on the intrinsic conductivity of its constituents. Polymer composites, on the other hand, are attractive alternatives since the insulating polymeric matrix can be highly flexible and optically transparent, whereas the electrical properties can be manipulated by controlling the architecture of conductive inclusions. The performance of the composite is then uniquely based not only on the intrinsic properties of its constituents but also on the arrangement of the conducting filler particles within the matrix and the contact between them. Although most physical properties of composite materials are generally enhanced by increasing the filler content, optical transparency tends to be reduced due to the difference in refractive index between filler particles and matrix. Thus, new approaches to fabricate flexible composite materials displaying both enhanced electrical conductivity and high optical transparency must be developed to fulfil the current requirements of optoelectronic applications.

Recent efforts have focused on employing conductive anisotropic particles exhibiting high aspect ratio to reduce the amount of opaque material needed to reach the percolation threshold in the composite^11^,^12^,^13^,^14^,^15^. Elongated allotropes of carbon such as multi- or single-wall carbon nanotubes have been used to yield electrical conductivity corresponding to a sheet resistance down to 290 Ohm sq^−1^ in cellulose nanofibrils aerogel membranes while maintaining 90% in transparency and 5% in strain at rupture^16^ or 34 Ohm sq^−1^ in 81% transparent polyethylene terephthalate-congo red single-wall carbon nanotube composites^17^. Graphene nanosheets are particularly promising in view of their unique mechanical and electronic properties^18^,^19^,^20^,^21^ and their availability through well-established exfoliation methods^22^,^23^,^24^,^25^,^26^. Nevertheless, even the remarkable percolation thresholds typically achieved in graphene-based composites are not low enough to yield materials with acceptable optical transparency and colour neutrality^27^. The use of the more processable graphene oxide may partially improve this scenario, yet reducing the conductivity, with the fate of the final material's optoelectronic properties still depending on the initial precursors formulation and the processing route followed^28^. Although highly conducting and transparent materials based on graphene have already been obtained using processing pathways such as transfer printing combined with thermal treatment^29^ or chemical vapour deposition followed by etching^30^,^31^, the fabrication costs associated with these techniques greatly reduce their potential for large-scale applications and call for new strategies.

One enticing approach to further decrease the amount of conductive particles needed to achieve the percolation threshold is to fabricate hierarchical composites exhibiting multiple percolating networks at different length scales^32^,^33^. While the potential of this approach has already been demonstrated for carbon nanotube-based composites^34^, translation of such concepts into industrially relevant processes requires simple and cost-effective strategies that allow for a deliberate control over the spatial distribution of the secondary network. Furthermore, combination of multiple percolation networks with sufficient optical transparency remains yet to be demonstrated.

In this study, we describe a simple processing route to fabricate transparent and conducting polymer-based composite films using external magnetic fields to assemble magnetized graphene flakes into hierarchical networks of predefined features. Using virtual magnetic moulds, transparency is obtained by locally concentrating the dark conductive flakes into continuous networks. When large micrometric mesh sizes are used as moulds, such control is achieved over areas spanning over several centimeters, resulting in double-percolating hierarchical networks. In addition to spatial control, tuning the orientation of the functionalized graphene flakes within the microscopic pattern further enhances the electrical conductivity. By carefully engineering the mesh size of virtual magnetic moulds, we demonstrate how this new strategy can lead to transparent, electrically conducting polymer–graphene composites with great potential for applications in advanced compliant optoelectronics.

## Results

### Synthesis and processing strategy

**Figure 1:**
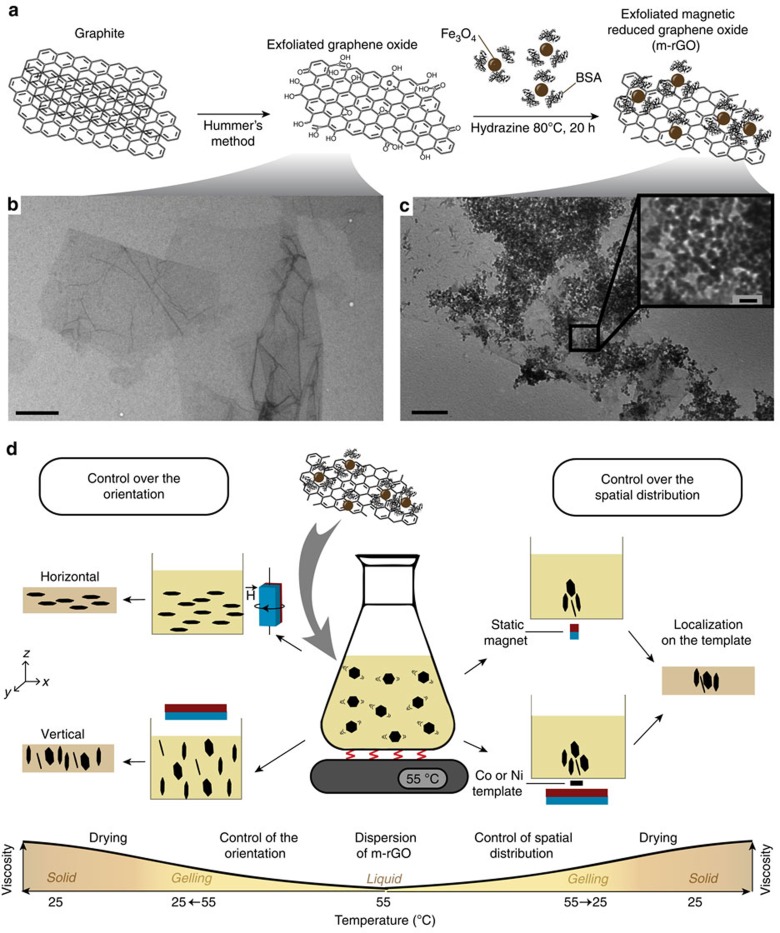
Preparation of graphene-based composite films with predefined architectures. (**a**) Synthesis of hydrophilic magnetically responsive reduced graphene oxide (m-rGO) mediated by bovine serum albumin (BSA). Transmission electron microscopic (TEM) images of (**b**) exfoliated graphene oxide and (**c**) m-rGO flakes ((**b**,**c**) Scale bar, 1 μm) with 10-nm diameter iron oxide nanoparticles (Scale bar, 200 nm (inset)). (**d**) Processing of gelatin-based composites with magnetic control over the orientation or spatial distribution of the m-rGO flakes. The magnetic assembly is performed in the liquid phase and is followed by consolidation of the matrix to yield composite materials with tailored structures at both nano- and microscales.

Hydrophilic magnetically responsive reduced graphene oxide (m-rGO) flakes are synthesized through decoration of exfoliated micrometric graphene oxide nanosheets (GO) with 10 nm superparamagnetic iron oxide nanoparticles (SPIONs; (Fig. 1a–c)). The attachment of SPIONs is assisted by a multidomain protein, bovine serum albumin (BSA), which maintains the magnetic nanoparticles adsorbed on the flake surface throughout all the preparation steps. The physical adsorption of the BSA generates a predefined molecular coating that settles the saturation limit to the number of adsorbed molecules and provides more available sites for the adsorption of SPIONs. After partial reduction by BSA, a treatment with hydrazine at 80 °C converts the GO into reduced graphene oxide (rGO)^35^ (see Supplementary Fig. 1 and Supplementary Discussion for details about reduction). If suspended in a liquid, the prepared m-rGO flakes exhibit high magnetic response, similar to the ultrahigh magnetic behaviour observed for other anisotropic particles decorated with SPIONs^36^. Remarkably, in contrast with the hydrophobic nature of rGO, the presence of polar organic groups in the surface-adsorbed SPIONs and BSA enables the successful dispersion of the m-rGO flakes in polar solvents, preventing the formation of possible defects associated with graphitic aggregation, clustering or deterioration of the matrix during post-reduction treatments^37^,^38^. We therefore take an advantage of this unusual hydrophilicity of the m-rGO flakes to incorporate them into two exemplary commonly used hydrogels, namely gelatin from bovine skin and poly(2-acrylamido-2-methyl-1-propanesulfonic acid) (PAMPS). These hydrogels exhibit a liquid-to-solid transition, which can be effectively used to fix the orientation and spatial distribution of the m-rGO flakes after the magnetically driven assembly process (see Fig. 1d, Supplementary Figs 2 and 3 and Supplementary Methods for details of the process and applicability of the strategy to polyurethanes).

The obtained m-rGO flakes can be deliberately aligned using low-cost rare-earth magnets. Biaxial horizontal alignment of the m-rGO flakes in the *xy*-plane is achieved by applying a horizontally rotating magnetic field^36^. Literature reports that vertical orientation of anisotropic particles is nontrivial and is usually achieved by laborious and high power energy means or other costly post processing techniques^39^,^40^,^41^,^42^. Here we deliberately orient the m-rGO flakes vertically by applying low static magnetic fields of 50 mT parallel to the *z* axis (Fig. 1d, left).

Besides orientation, the spatial distribution of m-rGO flakes can be controlled using magnetic fields exhibiting gradients in magnitude. Indeed, local gradients in magnetic field generate magnetophoretic forces that can efficiently attract suspended particles exhibiting magnetic susceptibility different from that of the surrounding liquid^43^. Graded magnetic fields can be easily designed using small permanent magnets, magnetically patterned stripes or virtual magnetic moulds comprising a metallic template, typically nickel or cobalt, placed above a larger permanent magnet (Fig. 1d right)^43^,^44^. All these methods enable scalable localization of m-rGO flakes in predetermined configurations and in time scales that cannot be achieved with 3D printing or other lithographic methods^9^,^45^.

### Orientation and spatial distribution control

**Figure 2:**
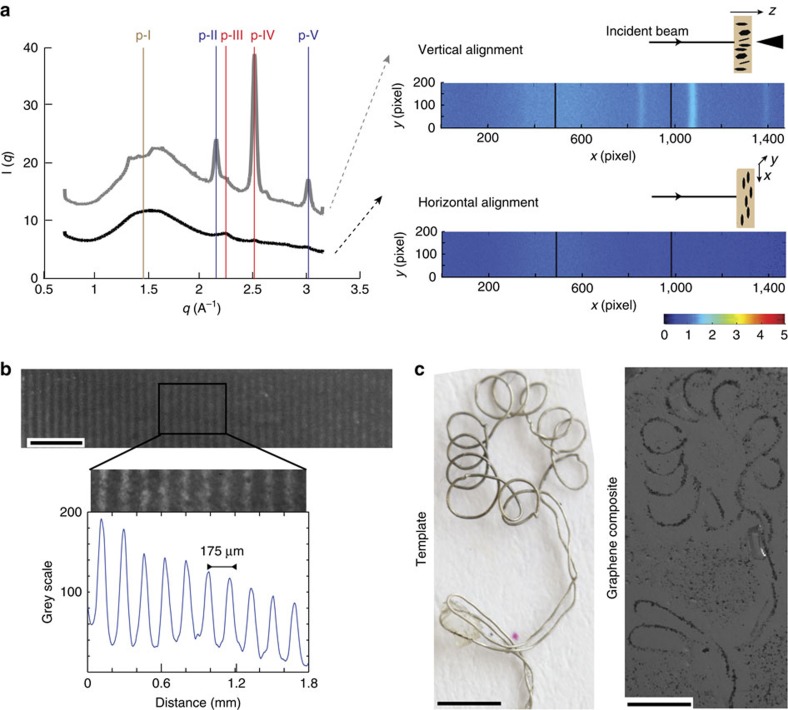
Hierarchically structured composites exhibiting controlled orientation and spatial distribution of m-rGO flakes. (**a**) Wide-angle X-ray Scattering (WAXS) of 0.96 vol% m-rGO–gelatin composites exhibiting horizontal (black) and vertical (grey) orientation of graphene flakes. Scattering peaks correspond to the gelatin matrix (p-I in brown), the SPIONs (p-II and p-V, blue) and the graphene (p-III and p-IV, red). (**b**,**c**) Spatial control of m-rGO flakes (**b**) within a gelatin matrix (7.84 vol% m-rGO) using a patterned magnetic stripe (Scale bar, 2 mm) and (**c**) within a PAMPS matrix (0.037 vol% m-rGO) using a virtual magnetic mould with a complex shape (Scale bar, 2 mm).

We demonstrate the ability to control the orientation and spatial distribution of m-rGO flakes by Wide- and Small-Angle X-rays Scattering measurements and optical microscopy (Fig. 2; Supplementary Fig. 4). The significant increase in the scattering intensity from composite films containing m-rGO flakes aligned parallel to the X-ray beam (along the *z* axis) compared with a horizontal configuration is the first evidence of the successful alignment of the flakes in the intended direction (Fig. 2a). In addition, in the vertical alignment, several intense Bragg reflections emerge, characteristic of either gelatin, rGO or SPIONs (see Supplementary Discussion), which cannot be resolved in the horizontal alignment.

In the two examples in Fig. 2b,c we used magnetic fields to drive the spatial distribution of graphene flakes within the *xy*-plane of the composite. In Fig. 2b, the m-rGO flakes are assembled within the gelatin matrix into a striated pattern using the low coercivity magnetic stripe of a standard train ticket. After consolidating the matrix, the resulting 30.7-μm-thick film exhibits features of similar dimensions as the magnetic stripe, with a periodicity of 175 μm between m-rGO-free and m-rGO-concentrated areas. To showcase the potential of using virtual magnetic moulds to obtain m-rGO-based composites exhibiting more complex microstructural designs, we fabricated a template using a 250-μm-thick nickel wire bended and curved into a flower-like shape. Placing the metallic template 1 mm above a permanent magnet of 250 mT, a virtual magnetic mould is generated, featuring magnetic field microgradients to guide the assembly of the m-rGO flakes in the shape of the template. The mirrored picture of the template and the optical microscopy image of the resulting 0.02 vol% m-rGO-PAMPS composite film confirm that shape and dimension of the virtual magnetic mould template are precisely translated to the m-rGO-based composite (Fig. 2c).

### Tuning transparency

**Figure 3:**
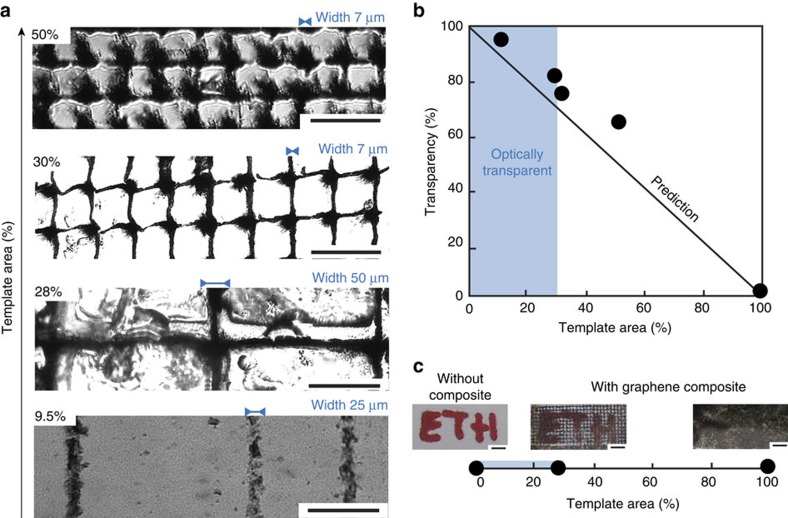
Optical transparency of hierarchical graphene-based composites assembled on mesh-like magnetic virtual moulds. (**a**) Optical micrographs of PAMPS composites containing 0.065 vol% m-rGO flakes assembled into mesh-like patterns of decreasing template area and different line width (Scale bar, 100 μm). (**b**) The transparency of the 30-μm-thick composite film containing 0.065 vol% m-rGO-PAMPS increases with decreasing coverage area of the templates. (**c**) Demonstration of the improved optical transparency by controlling the spatial distribution of m-rGO flakes within 0.75 vol% rGO–gelatin composite films using metallic templates of different coverage areas (Scale bar, 1 mm).

Positioning m-rGO flakes into two-dimensional (2D) patterns through magnetic manipulation of diluted dispersions enables the creation of hierarchical double-percolating networks (Fig. 3). At the nanoscale the network consists of an assembly of interconnected m-rGO flakes, whereas the 2D pattern gives rise to the second network at the microscale. Differently from the great majority of the approaches proposed in literature, such a directed hierarchical architecture is an efficient strategy to control the optical transparency of the composite film without altering its initial composition (see Supplementary Methods for more details about the definition of transparency adopted here). Similarly, colour neutrality can be preserved up to relatively high concentrations of graphene. To illustrate this approach, we designed a series of experiments where 0.065 vol% m-rGO flakes in PAMPS are localized into continuous mesh patterns of decreasing coverage area using 10-μm-thick nickel transmission electron microscopic grids, directly affecting the overall transparency of the composite film (Fig. 3a; Supplementary Fig. 5). The optical transparency of each composite shows a good agreement with the values expected for films with flakes covering precisely the templated area, confirming that the absolute template area controls the transparency of the composite, regardless of the line thickness of the template (Fig. 3b). The striking increase in optical transparency enabled by our approach is further evidenced by comparing composite films containing homogeneously distributed flakes with those fabricated using magnetic virtual moulds. For the same film thickness of 47±2 μm, 0.75 vol% rGO–gelatin composites with randomly distributed m-rGO flakes are dark and opaque, whereas films obtained by using a 28% area coverage template are 80% more transparent, allowing the observation of objects throughout the composite film (Fig. 3c).

### Combining transparency with electrical conductivity

**Figure 4:**
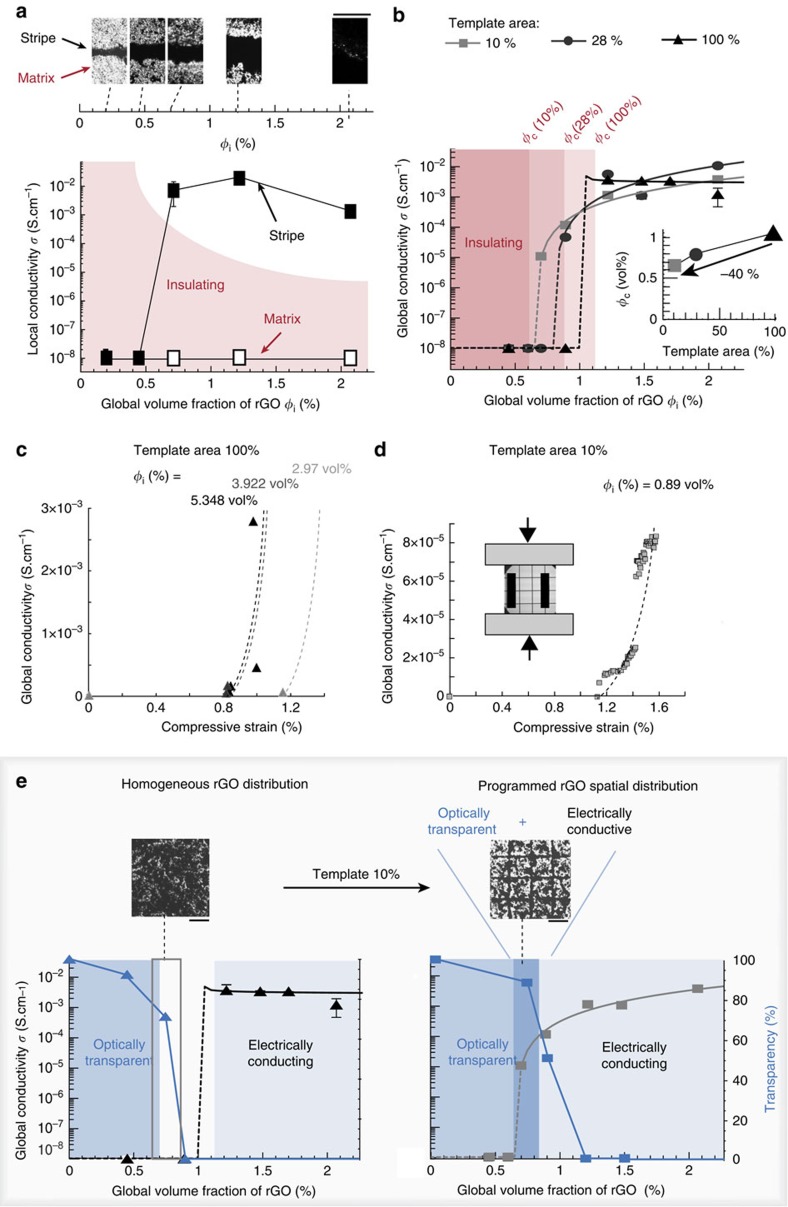
Electric response and transparency of gelatin composite films with magnetically driven percolation threshold. (**a**) Optical micrographs (Scale bar, 500 μm) and electrical conductivity measurements demonstrating the local control over electrical conductivity by assembling a volume fraction *ϕ*_i_ (vol%) of m-rGO flakes into a stripe in gelatin composite films (one hierarchical level). (**b**) Reduction of the percolation threshold *ϕ*_c_ as a function of the mould's template area in gelatin composite films (two hierarchical levels). Experimental global conductivity points are fitted with equation (1). (**c**,**d**) Evolution of the global conductivity under compressive strain for composite films prepared with a magnetic template area of 100% and 10%, respectively. The dotted lines are guides to the eyes underlining the trend. The inset in **d** shows a schematic drawing of the set-up, indicating the direction of the pattern and measuring electrodes relative to the applied force. (**e**) Controlling the rGO spatial distribution using a magnetic template of 10% area leads to transparent and electrically conductive gelatin films (dark blue region, right) of otherwise opaque and insulating homogeneous films for rGO global volume fractions of 0.65–0.85 vol% (region framed in grey, left). The optical micrographs were obtained from gelatin films containing 0.75 vol% rGO (Scale bar, 500 μm).

Locally concentrating the conductive rGO nanosheets into continuous mesh patterns not only enhances the overall optical transparency of the film but also creates electrically conductive paths that increase the global conductivity of the composite material (Fig. 4). To study this unusual combination of properties in details, we first investigate the local electrical conductivity of individual stripes made by magnetically driven localization of the m-rGO flakes (one hierarchical level, Fig. 4a). Gelatin-based composites with different global concentrations of graphene flakes concentrated in individual stripes were fabricated using a 100-μm-thick and 1-mm-wide cobalt ribbon as a virtual magnetic mould positioned 1 mm above a 250 mT permanent magnet. The global volume fraction of rGO, *ϕ*_i_ is calculated from the density of the m-rGO flakes and the coverage of iron oxide nanoparticles and BSA (details in Supplementary Fig. 6 and Supplementary Methods). The local electrical conductivity is measured by contacting two electrodes on the stripe or in the matrix area (see Supplementary Fig. 7 and Supplementary Methods). For global concentrations of 0.45 vol% or lower, the local volume fraction of graphene is not sufficiently high to form a percolating network and the composite remains insulating. Increasing the global concentration of m-rGO flakes to 0.75 vol% results in composites with conducting lines and insulating surrounding matrix. Thus, the minimum volume fraction needed to obtain a local percolating network lies between 0.45 and 0.75 vol%. To the best of our knowledge, this is also the first example of magnetized rGO composite exhibiting electrically conducting properties^46^,^47^,^48^.

The combination of individual conductive paths at the nanoscale into percolating patterns at a coarser length scale is ideal for the electrical conductivity of the resulting hierarchical composite films. Similar to 3D percolating networks formed in polymer melts^33^, localizing the conductive elements into continuous mesh-like patterns of different sizes leads to geometrically defined 2D hierarchical networks with lowered global percolating thresholds (two hierarchical levels, Fig. 4b). Global electrical conductivities of the hierarchical networks are measured by depositing a gold–palladium electrode spanning the longest dimension of the templates on the surface of the composite films directly facing the magnet. To rationalize the effect of the spatial distribution of m-rGO flakes on the electrical properties of the films, we applied the following percolation model^49^:


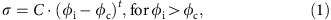


where *σ* is the global electrical conductivity, *ϕ*_i_ the global volume fraction of rGO in the composite film, *ϕ*_c_ the percolating threshold, *t* the conductivity exponent describing the percolation behaviour above threshold and *C* an empirical constant. This equation is valid for the overall single percolation as well as for multiple percolated systems, as demonstrated both theoretically and experimentally^32^,^33^. Fitting equation (1) to the experimental conductivity values confirms the expected percolating behaviour of the system above the threshold. Exponents *t* of 1.76 and 1.95 were obtained for composite films fabricated from templates with coverage areas of 10% and 28%, respectively. Such values are lower than the expected exponent of *t*_2D network_=2 × *t*_1D network_=2.7, where *t*_1D network_ is the exponent in the case of a single network, equal to 1.35 in this system^32^,^33^ (Supplementary Table 1). This probably results from the combined detrimental influence of the vertical orientation of the rGO nanosheets in composite films with higher volume fractions of particles with the non-conductive BSA/SPIONs coating of the nanosheets, reducing the number of contact points (see Supplementary Note 1 for details). Well-established approaches to remove surface-adsorbed species after assembly could be used to improve further the conductivity while maintaining the hierarchical magnetic assembly. Despite the lower exponent *t*, a remarkable decrease of 40% of the percolation threshold is observed for composite films containing m-rGO flakes spatially distributed over a template area of 10% (see Supplementary Fig. 8, Supplementary Table 2 and Supplementary Discussion for details on conductivity measurements on the opposite face of the films). This clearly demonstrates the potential of this method to tailor the percolation threshold of conducting anisotropic particles and to enhance the electrical conductivity of filler-loaded polymer films.

A possible further advantage of the proposed strategy is that of allowing the production of conductive and stretchable functional composites serving as strain sensors of high precision. Indeed, thanks to the intrinsic flexibility of polymers, we conducted in-plane confined compression experiments on polydimethylsiloxane substrates covered by the gelatin m-rGO film while recording the change in electrical conductivity (details in Supplementary Methods and Fig. 4c,d). As the compression strain increases, the graphene flakes are brought into closer contact, facilitating the electron transport and thus increasing the local conductivity. Remarkably, the benefits of the magnetically tuned percolation threshold become apparent by comparing the onset of the strain detection: a comparable detection limit of 1.1% strain is reached using just 0.89 vol% m-rGO flakes in films exhibiting 10% template area (Fig. 4d), as opposed to the 2.97 vol% needed in the case of a template area of 100% (Fig. 4c). In addition, above this detection limit, an increase of strain as small as 0.005% can be detected in 10% template area, in contrast with a minimum detectable strain of 0.16% for homogeneously distributed films (for *ϕ*_i_=5.35 vol%). Such a small resolution has not yet been reported in stretchable and transparent strain sensors used for health-monitoring devices where high strains, typically up to 300%, is the main property targeted^50^,^51^,^52^. In these systems, an error in strain of 2% is usually limiting the sensitivity^53^. Although other stretchable composites have been developed that reaches a detection limit down to 0.1% strain in carbon black filled rubber^54^ or 0.5% in graphene and silver nanoparticles sandwich structures^55^, they do not possess the optical transparency required for optoelectronic devices. The high sensitivity provided by our patterned composites indicates that the hierarchical network of m-rGO flakes not only decreases the global percolation threshold but also makes the conduction paths more strongly influenced by applied deformations. Furthermore, the formation of a hierarchical network of modified graphene flakes is also beneficial to keep the high deformability of the host matrix, which otherwise becomes brittle when the flakes are homogeneously distributed. These results underline the benefits that our strategy provides in adapting the mesh pattern geometry and the matrix composition for advances in the field of strain sensors, where high sensitivity is required in combination with the specific requirements and design found in health-monitoring systems and integrated electronic circuits.

Our ability to tune and control the spatial distribution of conductive paths as a means to combine optical transparency and electrical conductivity in the same composite is best illustrated when the obtained data are displayed simultaneously in a single plot of the relevant properties as a function of the global volume fraction of rGO (Fig. 4e). In composites containing homogenously distributed graphene flakes the global volume fractions required to achieve optical transparency and electrical conductivity are mutually excluded. Instead, using an initial template area of 10%, rGO–gelatin composite films containing 0.65 to 0.85 vol% of rGO can be made both transparent and conductive (Fig. 4e).

## Discussion

We have demonstrated that geometrically controlling the spatial distribution and orientation of magnetically functionalized graphene in polymer matrices is a simple and potentially up-scalable route for the design and fabrication of cost-effective, transparent and electrically conducting graphene-based functional composites. The design possibilities offered by the magnetic manipulation of graphene are mirrored by the great flexibility on the format of virtual magnetic moulds, allowing the creation of complex shapes with high efficiency and minimal efforts. Our results are complementary to existing approaches to design conductive and transparent materials, and prove the principle that conductive films can made transparent through magnetic assembly even if highly light absorbing constituents such graphene are used as the conductive phase. The implementation of such approach to the wide range of polymer films coated with metal nanowires and nanocarbon materials would enable a further increase in the electrical conductivity through the use of higher filler concentrations without compromising the film's optical transparency. Alternatively, the formation of a double percolation network of wires or nanotubes would enable the reduction of the filler content without sacrificing the electrical conductivity of the film.

This provides an exciting opportunity for the fabrication of next-generation stretchable optoelectronic sensors and devices that combine optimized design and materials meant to closely match target functions. Given the flexibility of the substrate, the known stretchability of graphene–polymer composites and the biocompatibility of graphene^56^,^57^, potential applications that could benefit from such systems range broadly from bio-integrated high-sensitivity strain sensors to high efficiency conformable solar cells.

## Methods

### Preparation of magnetic-reduced graphene oxide

Graphene oxide was prepared following a documented protocol^58^ (see details in the Supplementary Methods).

Spherical magnetite nanoparticles were synthesized by co-precipitation of FeSO_4_ and FeCl_3_ in the presence of NaOH. In a typical procedure, 0.25 mmol FeSO_4_·7H_2_O and 0.5 mmol FeCl_3_·6H_2_O (both from Sigma-Aldrich) were dissolved in 25 ml water and vigorously stirred. After heating to 60 °C, 10 ml NaOH solution at 2 wt% was added with 250 mg of hexadecylpyridinium bromide (Fluka).

A measure of 18 ml of the GO stock solution was mixed with 2 ml of 0.5 wt% of the BSA protein (Sigma-Aldrich). In a following step, this suspension was stirred with 250 μl of 4.4 wt% SPIONs suspension for 1 h to allow for the physical adsorption of the SPIONs at the surface of the graphene oxide sheets through interactions with the BSA. Volume of 100 μl of reducing agent hydrazine monohydrate (Sigma-Aldrich) was added at 80 °C for 20 h under continuous stirring. After cooling to room temperature, the mixtures were kept at 4 °C.

### Fabrication of m-rGO–gelatin composites

A preheated (55 °C, 30 min) aqueous solution of 20 wt% gelatin (from bovine skin, Sigma-Aldrich) was mixed with the corresponding volume of the suspension of m-rGO in water and stirred at 55 °C. For the experiments with controlled orientation, the mixture was cast into small polyethylene moulds. A static vertical magnetic field of 50 mT was used for the uniaxial alignment of the graphene flakes along the *z* axis, whereas the samples with biaxially aligned flakes were obtained by rotating the magnetic field on the *x*–*y* plane. The samples were afterwards cooled down to room temperature and dried for 24 h in ambient conditions to consolidate the gelatin and fix the designed architecture (Supplementary Fig. 2). Samples with controlled spatial distribution of m-rGO flakes were fabricated by casting onto a commercial magnetic stripe (train ticket) followed by cooling down and drying at ambient conditions (Fig. 2c). To demonstrate the decrease of the percolation threshold with the localization of the rGO, 250 μl of m-rGO–gelatin mixtures were cast into a well with bare surface, or with a nickel or cobalt template covered by a Teflon foil at the bottom (Supplementary Fig. 5). The wells were positioned on a permanent neodymium magnet of 250 mT (Supermagnete, Switzerland), before casting. The samples were then cooled to room temperature and dried in air, overnight.

### Fabrication of m-rGO-PAMPS composites

PAMPS hydrogel was synthesized according to a modified process^59^ (see Supplementary Methods for more details). The monomer solution was mixed with the relevant amount of solution of m-rGO and deposited in similar wells as described previously. The nickel template conformed in the shape of a flower was made by twisting a nickel wire. The trio magnet, well and suspension were placed in a dark chamber and irradiated by ultraviolet light (OmniCure Series 1000, Lumen Dynamics) for 2 min at 60% of the maximum power. The gelled composite films were then dried overnight at ambient temperature.

### Characterization methods

Characterization of the m-rGO and the composites film is detailed in the Supplementary Methods.

### Data availability

The data that support the findings of this study are available from the corresponding authors on request.

## Additional information

**How to cite this article:** Le Ferrand, H. *et al*. Magnetic assembly of transparent and conducting graphene-based functional composites. *Nat. Commun.* 7:12078 doi: 10.1038/ncomms12078 (2016).

## Supplementary Material

Supplementary Information Supplementary Figures 1-8, Supplementary Tables 1-2, Supplementary Note 1, Supplementary Discussion, Supplementary Methods and Supplementary References
