# Supplementary material for: Magnetic assembly of transparent and conducting graphene-based functional composites
Source: Nat Commun. 2016 Jun 29;7:12078. doi: 10.1038/ncomms12078 (PMC4931316; doi:10.1038/ncomms12078)
Supplement: Supplementary Information — Supplementary Figures 1-8, Supplementary Tables 1-2, Supplementary Note 1, Supplementary Discussion, Supplementary Methods and Supplementary References [file ncomms12078-s1.pdf]

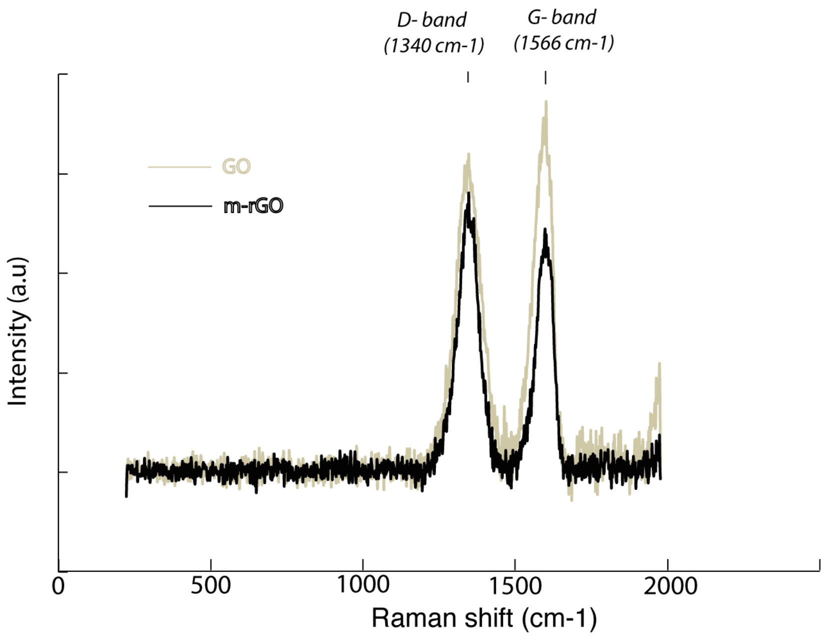

Supplementary Figure 1| Baseline-corrected Raman spectra of GO and m-rGO.

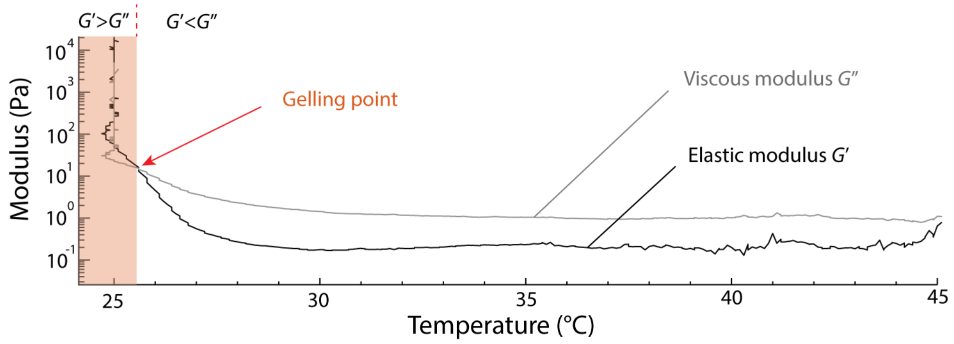

Supplementary Figure 2| Gelling of gelatin with temperature.

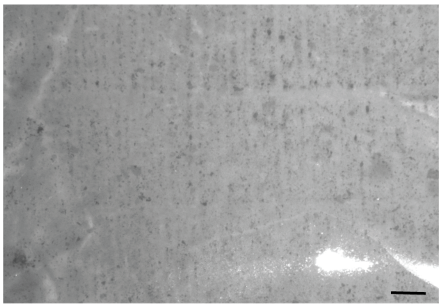

Supplementary Figure 3| Optical image of a polyurethane m-rGO composite film with localised m-rGO on a magnetic stripe (scale bar, 1 mm).

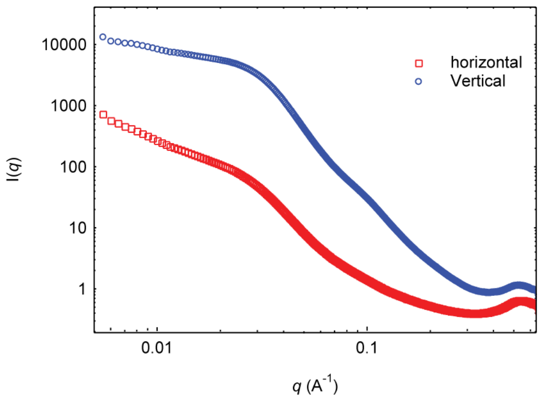

Supplementary Figure 4| SAXS of vertically and horizontally aligned m-rGO in gelatin (0.96 vol% rGO)

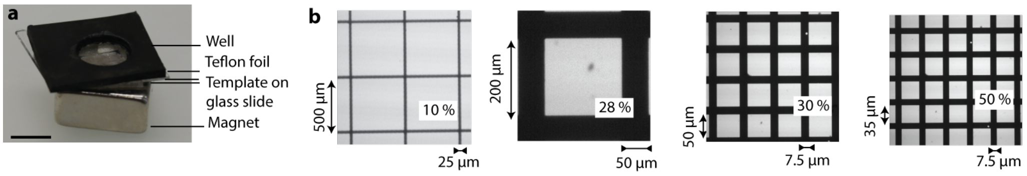

Supplementary Figure 5| a, Set-up for the concentration of m-rGO on a template (scale bar, 1 cm), **b,** Dimensions of the templates used with their template areas (%).

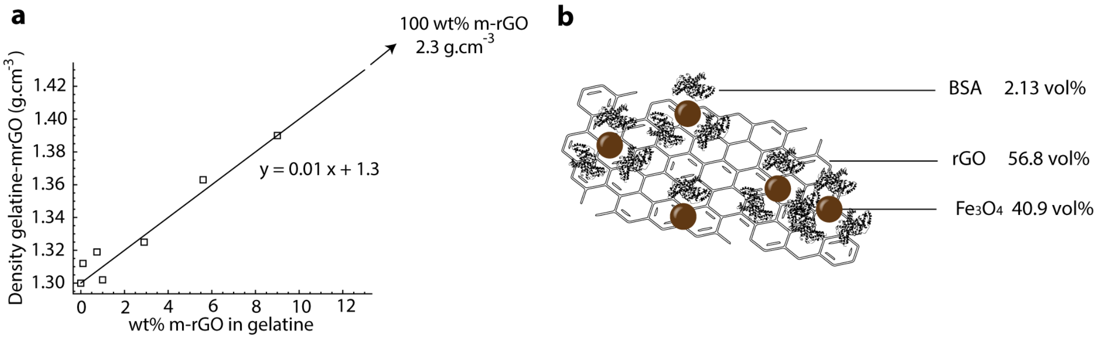

Supplementary Figure 6| a, Densities of the dried m-rGO-gelatin films and extrapolation to 100%. **b,** Schematics of one m-rGO flake with corresponding volume fractions.

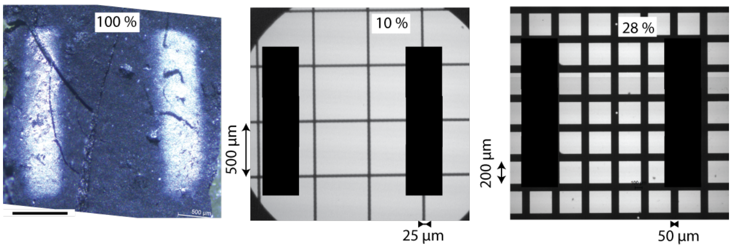

Supplementary Figure 7| Optical image of the electrode deposited on a 100% homogeneous m-rGO-gelatin composite film and schematics of the electrodes onto the 10% and 28% templates (scale bar, 500 µm, for the three images).

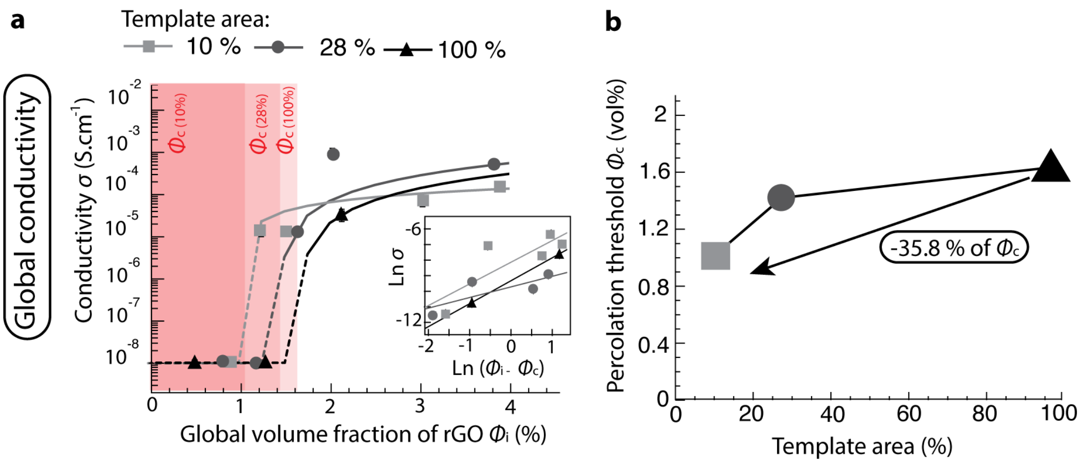

Supplementary Figure 8| Global conductivity of gelatin composite films with different localization areas measured on the top surface opposite to the magnet **(a)** with the fit with the equation 1 (main text) (inset) and the corresponding decrease in percolation **(b)**.

Supplementary Table 1: Fitting parameters of the global conductivity measured on the bottom surface: percolation threshold ϕ_c, exponent t and correlation coefficient between the fit and the experimental values R.

Pattern area	ϕ_c	Exponent t	R
100 %	1.054 vol%	-0.0673 (vertical alignment, see Supplementary Note)	0.941
28 %	0.795 vol%	1.9567	0.894
10 %	0.65 vol%	1.761	0.991

Supplementary Table 2: Fitting parameters of the global conductivity measured on the top surface

Pattern area	ϕ_c	Exponent t	R
100 %	1.644 vol%	1.3594	1
28 %	1.441 vol%	0.629	0.785
10 %	1.0548 vol%	1.2849	0.8187

Supplementary Note 1

We speculate that the negative exponent presented in Supplementary Table 1 for 100 % pattern area samples results from the vertical alignment of the m-rGO flakes. Indeed, at global volume fractions above 1.5 vol% steric hindrance and

jamming effects play a significant role, whereas low concentrations benefit from horizontal alignment. We observed that the vertically oriented field lines from the magnet placed underneath the sample resulted in the vertical alignment of the m-rGO flakes in the wet composite. Upon drying and shrinking, m-rGO flakes in the diluted composites are pushed into a horizontal configuration due to capillary forces developed during the removal of water. This effect is significantly reduced in composites with high graphene concentrations, in which steric hindrance and particle interlocking maintain the vertical orientation [1,2].

Supplementary Discussion

Raman characterization of reduced graphene oxide

A drop of respectively GO and of m-rGO suspensions was deposited on a glass slide and dried at 80 °C for 2 h. Each sample was analysed by using a confocal Raman microscope (InVia, Renishaw, UK) equipped with a linearly polarized laser at 532 nm and at a power of 0.8 mW. The Raman light was detected by an air-cooled CCD camera using integration times of 0.75 s and 1.5 s for GO and m-rGO, respectively. The spectra were baseline corrected using an intelligent fit provided by the software (InVia) (Supplementary Figure 1). The relative intensity between the two characteristic bands was for GO: $I_D/I_G = 0.88 \pm 0.05$ and for m-rGO: $I_D/I_G = 1.09 \pm 0.03$. This increase in the ration between the two bands indicates an increase in the presence of sp^2 carbon, thus of graphitic domains resulting from the reduction[9]. Moreover, the position of the G band is slightly shifted from 1566 cm^{-1} for the GO to 1578 cm^{-1} for m-rGO, thus approaching the value of natural graphite, 1581 cm^{-1} [10].

Wide and Small Angle X-ray Scattering (WAXS/SAXS) of aligned composite gelatin films

The multiple building blocks present in the composite lead to several scattering peaks for samples with horizontally or vertically-aligned m-rGO flakes. The large diffusive halo from the helical pitch or mean distance between neighboring peptide chains in gelatin is present in both spectra as the peak p-I at 4.6 $\dot{A}$ (q= 1.36 $\dot{A}^{-1}$) (see WAXS in Fig. 2a, main text)[3–6]. The inter molecular lateral packing of triple helices of gelatin can be found at a distance of 10-16 $\dot{A}$ (q= 0.39-0.62 $\dot{A}^{-1}$) in the SAXS spectra (see Supplementary Figure 4). Since graphene nanosheets are nearly perfect two-dimensional crystals, they also display specific Bragg diffraction peaks[7]. When the m-

rGO flakes are oriented along the z-direction (vertically, see Fig. 2a in the main text for the definition of the axis), reflections from the lattice planes become dominant giving rise to the Bragg peaks p-III at 2.21 $\dot{A}^{-1}$ and p-IV at 2.56 $\dot{A}^{-1}$ (see WAXS in Fig. 2a, main text). Such peaks correspond to the characteristic distances among carbons within the graphene, namely 2.84 $\dot{A}$ (p-III) for the C_1-C_4 distance and 2.45 $\dot{A}$ (p-IV) for the C_1-C_3 distance. Both these lengths are very close to the corresponding theoretical values obtained from a perfect honeycomb chemical structure of carbon in sp^2 hybridization. The other two peaks emerging when m-rGO flakes are aligned along the z-direction, referred to as p-II at 2.12 $\dot{A}^{-1}$ and p-V at 2.99 $\dot{A}^{-1}$ (see WAXS in Fig. 2a, main text), are tentatively attributed to Bragg reflections from the (220) and (400) planes of the SPIONs, respectively[8]. Conversely, composite films containing m-rGO flakes oriented in the xy-plane show rather weak Bragg reflections at these *q* values specific of SPIONs. Remarkably, the absence of the graphitic peak at 3.35 Å (q= 1.875 $\dot{A}^{-1}$) and 7.9 $\dot{A}$ (q= 0.8 $\dot{A}^{-1}$) in both situations confirms the successful exfoliation and stabilization of rGO into separated single nanosheets through surface modification using BSA and SPIONs (see WAXS in Fig. 2a, main text). Finally, the shoulder in the SAXS measurements at 0.03 $\dot{A}^{-1}$ reflects the 20 nm iron oxide nanoparticles.

Global conductivity and decrease of the percolation threshold in gelatin m-rGO composite films measured on the face opposite to the magnet

The global conductivity of the composite films with localized m-rGO on templates of 10, 28 and 100% area was measured on both the bottom and top surfaces. The main manuscript gives the results for the bottom surface lying close to the magnet. The opposite surface further away from the magnet (top) exhibits the same decrease in the percolation threshold but with threshold values shifted towards higher volume fractions. Indeed, the magnet being closer to the bottom surface, magnetophoretic forces are directed towards this surface. Nevertheless, these series of measurements confirm the decrease in percolation threshold when concentrating rGO on continuous mesh patterns (Supplementary Figure 8). Supplementary tables 1 and 2 summarized the results for both bottom and top measurement. The percolation threshold can be measured directly form the conductivity curves, while the exponents *t* are obtained by fitting the experimental data points according to the percolation theory (Equation 1, main manuscript).

Supplementary Methods

Synthesis of graphene oxide (GO)

Graphene oxide was prepared following a documented protocol[11]. A mixture of 3.7 g of graphite powder (99.5% purity, Graphit Kropfmühl) and 120 mL of concentrated sulphuric acid (98.5%) was prepared and stirred in an ice bath. Oxidation took place after addition of 2.5 g of $NaNO_3$ followed by 11.6 g of $KMnO_4$. The temperature was carefully kept under 20 °C to counteract the exothermic reaction. The mixture was then continuously stirred for 7 hours at 35 °C before further addition of 9.5 g of $KMnO_4$ and 14 hours stirring at 35 °C. The reaction was stopped by pouring the mixture on 600 mL of distilled water under stirring. A change of color from dark to brownish occurred after addition of 20 mL of 30% H_2O_2 indicating the full oxidation of the graphite. The solution was purified after 10 cycles of centrifugation (3000 rpm, 10 min) and washed with 5% HCl (Sigma-Aldrich) in an ultrasonic bath. After 3 final washing steps in ethanol, the solid powder was dried at 60 °C overnight. The powder was then mixed in water at a concentration of 0.7 wt% and ultrasonicated for 1 h (Ultrasonic cleaner, VWR). After centrifugation, the supernatant containing the stabilized dispersion of GO was collected and dialyzed (ZelluTrans ROTH, MWCO 4.000-6.000, Roth) in 4 L of milliQ water (Nanopure Diamond, Barnstead, Switzerland) to remove the remaining ions. The conductivity of the final solution was 554 $\mu S.cm^{-1}$.

TEM characterization of the m-rGO complex

Transmission electron microscopy (TEM) images were obtained by a Philips TEM (CM 20) instrument operating at a voltage of 100 kV. A drop of the suspension of the m-rGO in water was cast onto a carbon support film on a copper grid. The excess solution was removed after 30 s by blotting using a filter paper.

Consolidation of gelatin m-rGO composite films with controlled orientation or localization

Supplementary Figure 2 shows the consolidation of gelatin when cooling down to room temperature. Rheological measurements were carried out in a Gemini rheometer (Bohlin) with a cone-plane geometry and in controlled stress mode. The viscosity of pure gelatin was measured after warming the cone and plate measuring

tool at 50 °C prior to the test. Oscillation rheology at a constant frequency of 1 Hz and constant stress of 10 Pa was used to measure the elastic and viscous moduli.

To demonstrate the decrease of the percolation threshold with the localization of the rGO, 250 µL of m-rGO-gelatin mixtures were cast into a well with bare surface, or with a nickel or cobalt template covered by a Teflon foil at the bottom (see Supplementary Figure 5). The wells were positioned on a permanent neodymium magnet of 250 mT (Supermagnete, Switzerland) prior to casting. The samples were then cooled to room temperature and dried in air overnight.

Preparation of polyurethane m-rGO composite film

Water from the mother solution of m-rGO could be replaced by dimethylformamide (DMF, 99,8%, Sigma-Aldrich) by a solvent exchange method using a rotavapor (Butch, Switzerland). This enables the selective evaporation of water after addition of the same amount of DMF to the initial solution. This new suspension was mixed to a solution of 3 wt% of polyurethane (Elastollan, C64 53000) in DMF and casted on a commercially available magnetic stripe. After total evaporation of the DMF at 60 °C overnight, the polyurethane film exhibited the same striated pattern as in the magnetic stripe (Supplementary Figure 3).

Wide and Small Angle X-ray Scattering (WAXS/SAXS) of the composite films

WAXS / SAXS measurements were performed at the coherent Small-Angle X-ray Scattering (cSAXS) beamline at the Swiss Light Source (Paul Scherrer Institute, Switzerland). The X-ray wavelength of 0.1 nm was obtained from a double-crystal Si (111) monochromator and focused using the bendable monochromator crystal and high-order rejection mirror. 2D diffraction patterns were recorded with the PILATUS 2M detector developed at PSI. The total sample to detector distance (7.158 m) and the center of the direct beam on the detector were calibrated using silver behenate.

Optical characterization and transparency measurements

Optical microscopy images were taken either with a small inverted microscope (Leica DMIL LED, Camera Leica DFC295, Switzerland, Fig. 3a and 4a,b main manuscript), a travelling macroscope (Leica Z16 APO, Camera Leica DFC 365 FX, Switzerland, Fig. 2c) or a camera (Fig. 2d and 3c, main manuscript). The profile plot of the striated gelatin composite film was extracted using ImageJ (Image J 1.47v). The transparency of the composites was calculated as the ratio of the grey value of the whole image of the composite film, I_2, divided by the average grey value of the

polymer matrix, I_1. The grey values were extracted using ImageJ. According to Paine *et al*[12] a material is said to be highly transparent if it shows a transparency of 90% or more at 100 nm thickness. Using the Beer Lambert law (Supplementary Equation 1 in the following) relating the incident light I_0 and transmitted light I_1, this would mean that a film is defined as "optically transparent" if

$$I_1/I_0=e^{-a_1 x} > 0.9 \qquad (1)$$

where x is the 100 nm thickness and a_1 is the attenuation coefficient of the material, in the present case, the native gelatin. By applying the supplementary equation 1, this would mean that an attenuation coefficient a_1 inferior to 10^{-3} nm^{-1} is required to have a transparent material.

We now take the measured I_1 as the reference system (gelatin), to detect what is the reduction in transparency after producing double percolation grids. After confining graphene in double percolating networks, the light I_2 which goes through a sample of thickness x is:

$$I_2/I_1=e^{-a_2 x} \qquad (2)$$

where a_2 is now the attenuation coefficient of the meshed gelatin containing the double percolating network. Taking for I_2/I_1 the value of 0.85 measured for our 30 µm thick film, leads to an attenuation coefficient a_2 of 5×10^{-6} nm^{-1} which is at least three orders of magnitude lower than the value of 10^{-3} nm^{-1} used above to define transparency.

We then have for the true transparency of the final material:

$$I_2/I_0=(I_2/I_1)*(I_1/I_0)= e^{-a_2 x} * e^{-a_1 x} = e^{-(a_1+a_2)x} \qquad (3)$$

The new attenuation coefficient of the whole composite film is therefore $a_1 + a_2$, but while a_1 is intrinsic to the continuous matrix, a_2 is typical of the procedure followed here. In other terms, it is a_2, which quantifies the relative transparency between the original material and the same material after modification with the double percolating mesh network. We further note that a_1 can be chosen to be virtually equal to 0 (using a 100% transparent polymer), making relative transparency equivalent to true transparency.

Determination of m-rGO density

The volume fraction of m-rGO within the dried composite gelatin film was calculated by extrapolating the densities of m-rGO-gelatin composites of known composition to 100% m-rGO (Supplementary Figure 6). The densities were measured using the Archimedes principle and hexane (96%, Mutlisolvent, Scharlau, Spain) as a solvent. This revealed that the density of the m-rGO, ρ_{mrGO}, is 2.3 g.cm^{-3}. Additionally, given the known amount of 10 nm spherical iron oxide particles and

BSA adsorbed to the rGO and their respective densities of 5.24 and 1.22 $g.cm^{-3}$, the iron oxide nanoparticles and BSA coating can be estimated to represent 40.9 vol% and 2.13 vol% of the total volume of the modified flakes, respectively. Therefore the volume fraction in rGO within the final dried gelatin composite film was calculated based on the initial amount of m-rGO, m_{mrGO}.:

$$V_{\mathrm{mrGO}} = \frac{m_{\mathrm{mrGO}}}{\rho_{\mathrm{mrGO}}} \tag{4}$$

$$V_{\mathrm{rGO}} = \frac{100-40.9-2.13}{100}\, V_{\mathrm{mrGO}} \sim \frac{V_{\mathrm{mrGO}}}{2} \tag{5}$$

$$Vol\%_{\mathrm{mrGO/gel}} = \frac{V_{\mathrm{mrGO}}}{V_{\mathrm{gel}}+V_{\mathrm{mrGO}}} \tag{6}$$

$$Vol\%_{\mathrm{rGO/gel}} = \frac{Vol\%_{\mathrm{mrGO/gel}}}{2} \tag{7}$$

Measurement of the local conductivity

The prepared gelatin-mrGO composite films were always thoroughly dried under 0.02 mbar (using Bal-Tec SCD 050 Sputter Coater under Argon flow) prior to electrical conductivity measurements. Macroscopic 2-points probe measurements were performed using a handheld/portable digital multimeter (Voltcraft VC230) while microscopic 2-points probe measurements were done using a Hall effect measurement system of Accent HL5510PC (cleanroom facility FIRST). The resulting conductivity was calculated with:

$$\sigma = \frac{d}{Rtl}, \tag{8}$$

where d = 1 mm is the distance between the electrodes, l = 250 µm is the average diameter of each electrode and t = 30 µm is the thickness of the composite film.

Measurement of the global conductivity

Masks with openings defining the geometry of the electrodes were prepared and taped onto the samples (Supplementary Figure 7). The electrodes were made by sputtering 37.5 nm of Au/Pd on the specimen surface. The resistance R between the electrodes was measured using a handheld digital multimeter. The resulting conductivity was calculated with:

$$\sigma = \frac{L}{RtW}, \tag{9}$$

where L = 900 µm is the distance between the electrodes, W = 1190 µm is the width of each electrode and t = 30 µm is the thickness of the composite film.

Variation of the electrical conductivity with applied strain

The gelatin m-rGO composite films were coated by a 3 mm-thick layer of polydimethylsiloxane (PDMS, Sylgard 184 Silicone Elastomer kit, Dow Corning, Michigan, USA) and cured for 1 hour at 100 °C. No high vacuum was applied to the samples to maintain the flexibility of the substrate. This explains the higher resistivity obtained when compared with gelatin composite films subjected to vacuum. Copper wires are then inserted throughout the sample at the places of the electrodes and wired to the multimeter. The specimen was then placed in a mechanical tester (Shimadzu, TeMeCo, Switzerland) mounted with a confined compression set-up. Both strain and electrical resistance are recorded during the mechanical test. The values of the conductivity are calculated as described previously, taking into account the deformation of the electrodes.

Supplementary References

1. Yousefi, N. *et al.*, Simultaneous in situ reduction , self-alignment and covalent bonding in graphene oxide / epoxy composites. *Carbon* **59**, 406–417 (2013).

2. De Volder, M. & Hart, A. J. Engineering hierarchical nanostructures by elastocapillary self-assembly. *Angew. Chemie* **52**, 2412–2425 (2013).

3. Balian, G. & Bowes, J. H. The structure and properties of collagen. *The Science and Technology of Gelatin, Academic Press: London*, 1-27 (1977).

4. McLaughlin, G. D. & Theis, E. R. The chemistry of leather manufacture, Reinhold Publishing Corporation, New York (1945).

5. Harrington, W. F. & Von Hippel, P. H. The structure and properties of collagen and gelatin. *Adv. Protein Chem.* **16**, 87–98 (1961).

6. Pezron, I., Djabourov, M., Bosio, L. & Leblond, J. X-ray diffraction of gelatin fibres in the dry and reswollen states. *J. Polym. Sci. part B-Polymer Phys.* **28**, 1823–1839 (1990).

7. Meyer, J. C. *et al.*, The structure of suspended graphene sheets. *Nature* **446**, 60–63 (2007).

8. Teymourian, H., Salimi, A. & Khezrian, S. Fe_3O_4 magnetic nanoparticles/reduced graphene oxide nanosheets as a novel electrochemical and bioeletrochemical sensing platform. *Biosens. Bioelectron.* **49**, 1–8 (2013).

9. Wang, H. *et al*., Solvothermal reduction of chemically exfoliated graphene sheets. *J. Am. Chem. Soc.* **131**, 9910–9911 (2009).

10. Wang, H. *et al*., Vibrational properties of graphene and graphene layers. *J. Raman Spectrosc.* **40**, 1791–1796 (2009).

11. Li, C., Adamcik, C. & Mezzenga, C. Biodegradable nanocomposites of amyloid fibrils and graphene with shape-memory and enzyme-sensing properties. *Nat. Nanotechnol.* **7**, 421–427 (2012).

12. Paine, et al., Gregory Crawford, *Technology & Engineering Book,* **Ch.5**, (2005).
